# An Overview of Strategies for Detecting Genotype-Phenotype Associations Across Ancestrally Diverse Populations

**DOI:** 10.3389/fgene.2021.703901

**Published:** 2021-11-05

**Authors:** Irving Simonin-Wilmer, Pedro Orozco-del-Pino, D. Timothy Bishop, Mark M. Iles, Carla Daniela Robles-Espinoza

**Affiliations:** ^1^ Laboratorio Internacional de Investigación sobre el Genoma Humano, Universidad Nacional Autónoma de México, Campus Juriquilla, Queretaro, Mexico; ^2^ Biostatistics Department, University of Michigan, Ann Arbor, MI, United States; ^3^ Leeds Institute for Data Analytics and Leeds Institute of Medical Research at St. James’s, University of Leeds, Leeds, United Kingdom; ^4^ Wellcome Sanger Institute, Hinxton, Cambridge, United Kingdom

**Keywords:** GWAS, admixture, ancestry, PCA, regression

## Abstract

Genome-wide association studies (GWAS) have been very successful at identifying genetic variants influencing a large number of traits. Although the great majority of these studies have been performed in European-descent individuals, it has been recognised that including populations with differing ancestries enhances the potential for identifying causal SNPs due to their differing patterns of linkage disequilibrium. However, when individuals from distinct ethnicities are included in a GWAS, it is necessary to implement a number of control steps to ensure that the identified associations are real genotype-phenotype relationships. In this Review, we discuss the analyses that are required when performing multi-ethnic studies, including methods for determining ancestry at the global and local level for sample exclusion, controlling for ancestry in association testing, and post-GWAS interrogation methods such as genomic control and meta-analysis. We hope that this overview provides a primer for those researchers interested in including distinct populations in their studies.

## 1 Introduction

Genome-wide association studies (GWAS) aim to identify genetic variants (usually single-nucleotide polymorphisms or SNPs) that are associated with a phenotype of interest. GWAS have been highly successful at identifying genetic variants influencing a large number of traits, with nearly 5,000 publications and more than 250,000 variant-phenotype associations included in the GWAS Catalog (Buniello et al., 2019). Not only have GWAS improved our understanding of the aetiology of complex traits, identifying potential new biological pathways influencing phenotypes, but they are also of potential clinical value in assessing an individual’s risk of developing particular phenotypes (e.g., Manolio (2013); Khera et al. (2018); Lambert et al. (2019)).

However, focusing only on participants of European descent, a characteristic of many published studies, restricts extrapolation to those of non-European ancestry (most notably for individual risk prediction (Mills and Rahal, 2019)) and limits available samples for traits common to multiple ancestries. By including populations with differing ancestries, the potential is enhanced for identifying causal SNPs or haplotypes because of the differing patterns of linkage disequilibrium (LD) across subpopulations. Driven by the need to identify SNPs with even more modest effect sizes to further elucidate genetic architecture, GWAS sample sizes have necessarily increased; therefore, studies of a wider range of populations are warranted. In recognition of this, the proportion of studies including individuals of non-European descent has increased in recent years (Gurdasani et al., 2019). Such adaptations of study design require re-assessment of analytical approaches; when individuals from multiple distinct genetic ancestries are included in a study, it is necessary to implement a number of control steps to ensure that the associations identified are not detecting ancestry-driven rather than trait-related genetic effects.

One of the challenges of performing association tests on genomic data is that demographic history influences the genomic structure of the population being analysed. If this is not properly controlled for, any genotype-phenotype association found in the study may be a consequence of this structure, rather than genuine trait association. The source of this potential bias is known as population stratification, where different trait distributions within genetically distinct subpopulations will result in those markers associated with the ancestry of the subpopulation to be also apparently associated with the trait. As an illustrative example, Choudhry et al. (2006) analysed the relationship between ancestry-informative markers (SNPs with considerably different allele frequencies between Native American, African, and European ancestral populations) and asthma. They found that three of the 44 tested markers appeared to be related to the disease in Mexicans, but none of these associations persisted when ancestry was controlled for suggesting that the association is driven at least in part by ancestry. Therefore, it is of utmost importance to ensure that either all the individuals in a study are from the same ancestry prior to performing a GWAS or that this ancestry is appropriately taken into account in the analysis.

Depending on the populations being studied, analysis may not be as simple as identifying subpopulations in the samples, since each individual may be descended from multiple subpopulations tracing back to a mixture event (or admix event) between them. One of the ways in which we can express this mixing in an individual is as a function of ancestral populations; that is, populations that have been isolated from each other in the past (e.g., European and African). If the combination of these ancestral populations has been recent, then we expect to observe longer LD tracts; but these will decay over time (Montana and Pritchard, 2004), thus adding to the complexity of finding significant relationships. However, the more diverse linkage disequilibrium structure also gives the possibility of finding more nuanced, ancestry-specific signals in a GWAS. The purpose of this review is to discuss the main approaches that are used in order to account for population structure in admixed individuals in a GWAS to select data to include, control for its influence on findings, and compare or aggregate results across populations.

In order to provide an understanding of the methods used for the analysis of admixed populations, we will first review the steps involved in performing a GWAS. Secondly, we will discuss some of the methods used in recent years to study admixed populations, and the way in which each methodology has been applied. Here, we will both explain the rationale behind each methodology and give some examples of applications in recent studies.

## 2 Controlling for Population Structure in Genome-Wide Association Studies

For the purposes of this review, we will divide a GWAS into three steps:

1) Quality control. (QC). This first, critical step involves filtering poor quality germline DNA samples and inconsistently performing SNPs from further consideration. This consists on applying specific filtering criteria to samples and/or SNPs before proceeding.
2) Association testing. Once QC has been completed, a statistical test is performed with the aim of detecting association between variants in the genome and the trait under consideration.
3) Post-GWAS interrogation. Once candidate SNPs have been identified, other types of analyses are performed to ensure the integrity of the association testing including that the influence of genetic structure has been well controlled for and to explore the characteristics of the SNPs identified including for instance biological processes implicated.

In steps 2 and 3, there are ways in which population structure can be taken into account, but it is important to note that we can use more than one technique on a single GWAS; in fact, they are often combined to avoid spurious associations.

In order to illustrate the use of these methods, we sampled data using the 1,000 Genomes Project (Consortium, 2015) dataset. We decided to use this dataset because of the self-reported ancestry label of the samples; these are useful for visualizing and comparing different methods.

## 3 Estimating Population Structure

The next subsection will cover two methods that are helpful in investigating the ancestry for each of the individuals in our data. These methods will be present throughout the review and will become useful for both quality control and genotype-phenotype association testing. The first one is admixture analysis, which assumes the existence of discrete ancestral populations from which the current population is derived. The second is principal component analysis, which generates explanatory variables from the genotype data that summarise the sources of variation among the samples and helps visualise and interpret the genetic structure of the samples.

### 3.1 Ancestry Estimation

Ancestry estimation aims to divide an individual’s genome between multiple ancestral populations from which it is hypothesised to have descended. Most methods used here follow a clustering approach, where each allele is assumed to have a probability of coming from one of the ancestral populations; these methods involve assessment of a large number of SNPs to estimate the contributions of each ancestral population. It is important to differentiate between two distinct forms of ancestry estimation: global and local (Thornton and Bermejo, 2014). Local ancestry is based on the fact that genetically adjacent regions form haplotypes whose ancestry can be probabilistically aligned to each population. There are local ancestry methods based on a model of recent admixture, and others that can infer gene flow from ancient hominids (Sankararaman et al., 2016; Durvasula and Sankararaman, 2019; Hubisz et al., 2020). The aim of global ancestry is to estimate the contribution, overall, of the genome from each ancestral population rather than each precise genomic region.

#### 3.1.1 Global Ancestry

The main assumption for this estimation is that a given individual is descended from ancestors drawn from distinct ethnic groups. The result of an analysis of this kind is an estimation of the proportion of each individual’s genome that comes from each of the ancestral populations.

**FIGURE 1:**
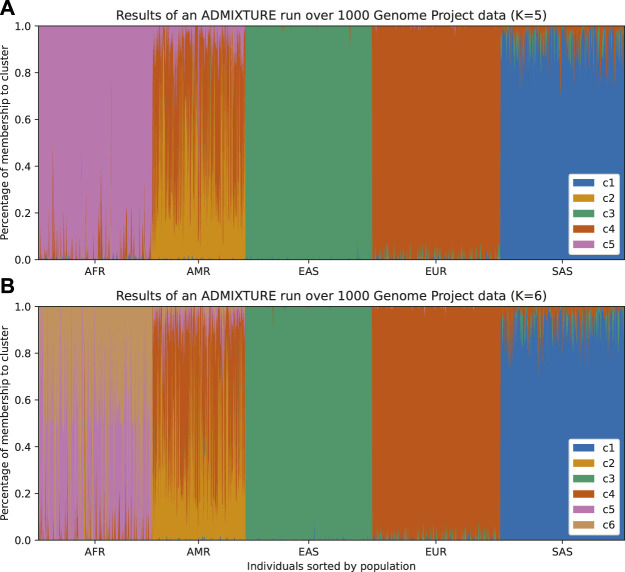
Individuals from 1,000 Genomes Project are plotted according to their labeled self-reported ancestry (AFR, African; AMR, Ad Mixed American; EAS, East Asian; EUR, European; SAS, South Asian). **(A)** Results from an ADMIXTURE analysis with K = 5 (number of clusters). The colors represent the clusters inferred from the data. In this figure, we can infer that c1 corresponds to South Asian ancestry, c3 to East Asian ancestry, c4 to European ancestry, and c5 to African ancestry. The Admixed American population appears as the most varied across clusters and has an exclusive cluster (c2), which suggests that there is a mix of *native* ancestry and influx from Africa and Europe. **(B)** By running ADMIXTURE with K = 6 we can appreciate similar results. The extra cluster indicates further structure within the African population, which could be either from admixture or the existence of subpopulations in the African samples, but the rest remains unchanged.

**FIGURE 2:**
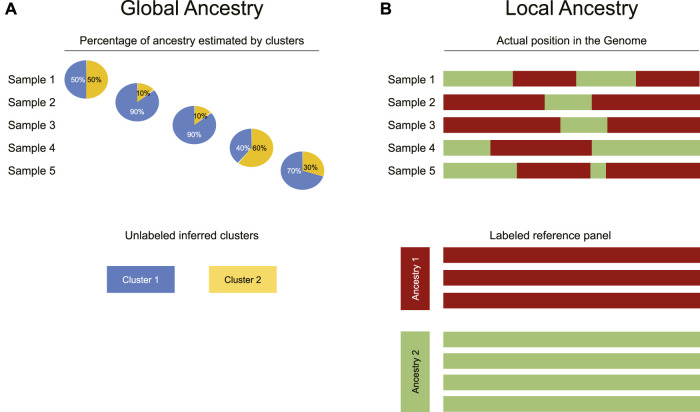
Differences between global and local ancestry analysis. **(A)** Global ancestry analysis infers the proportion of each individual’s genome that comes from each ancestral population (represented as clusters identified in an unsupervised manner). **(B)** In contrast to global ancestry analysis, local ancestry inference uses a reference panel to attribute each physical segment of the genome to a specific ancestry reported in the panel.

The two most popular algorithms for global ancestry calculation are STRUCTURE (Pritchard et al. (2000); Falush et al. (2003); Porras-Hurtado et al. (2013)) and ADMIXTURE (Alexander et al., 2009). Both of these algorithms require choosing the number of ancestral populations a priori and modeling the probability of membership to each ancestral population. STRUCTURE assumes a Bayesian model that accounts for linkage disequilibrium within each ancestral population, whereas ADMIXTURE assumes linkage equilibrium and uses the unlinked SNPs to apportion ancestry; this is a practical observation since an extra step will be required to run ADMIXTURE by thinning the SNPs to create this set of “independent” SNPs. The results can be visualized in an admixture plot, which shows the percentage of each subpopulation (given by the cluster) that the model assigns to each individual in the sample (Figure 1, and Figure 2A). While these methods return “estimates” of ancestry, care must be taken not to overinterpret these results in terms of alignment with population history.

#### 3.1.2 Local Ancestry

Although global ancestry uses unsupervised methods such as clustering, local ancestry is more restricted as it requires a locally recruited reference panel, enabling the estimation of the locus-specific likelihood of ancestry. In other words, for each SNP, the ancestral population from which it has most probably been inherited is calculated (Figure 2B). If the estimation is correct, this analysis achieves global ancestry estimation too.

Although there are several packages to infer local ancestry, there are two that are most commonly used. The first one is RFMix (Maples et al., 2013), which adjusts samples to a reference panel of known ancestries through a random forest procedure. The second algorithm is implemented in the software LAMP-LD (Baran et al., 2012), which uses Hidden Markov Models to relate the linkage disequilibrium in the population to a set of reference haplotypes.

### 3.2 Principal Component Analysis

Principal Component Analysis (PCA) is a dimensionality reduction method that finds the directions in the variable space under study that explain the most variance; these directions are called the components. In the case of genotype data, each SNP can be represented with values 0, 1 or 2 depending on the dosage of the alternative allele (aa, Aa, AA respectively, with “a” referring to the reference allele and “A” to the alternative). In this way, a data matrix can be created that has individuals in rows and SNPs in columns. From this matrix, we can compute the components. Each component is orthogonal to the others so they can be used, for example, to visualize the highly dimensional genotype data used in GWAS.

**FIGURE 3:**
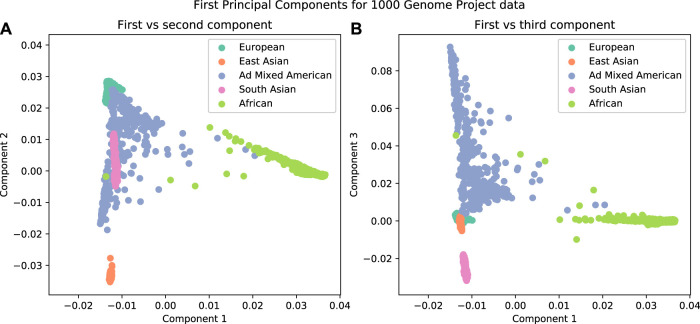
The first three components from a Principal Component Analysis on data from the 1000 Genomes Project. A clear separation is observed when plotting the individuals by their components. **(A)** First vs second components. **(B)** First vs third components.

It has been observed that the first few principal components from genotype data are related to population structure (Figure 3). The advantage of using this method over admixture analysis is that PCA results in a more nuanced view of the genetic structure of the sample, given that there is no need to specify the number of ancestral populations. A number of distinguishing characteristics can be appreciated when 1,000 Genomes data are plotted in this way; for example, the admixed American population overlaps with other populations in the first two principal components; this illustrates the admixture in those individuals (Figure 3A). But if further components are examined (Figure 3B), there is a clear separation of the American population from others.

PCA is a widely used method in different disciplines, so its implementations are abundant. Some of the more popular software for genotype data are the PLINK (Purcell et al., 2007) --pca method, EIGENSOFT (Price et al., 2006), and the SNPRelate (Zheng et al., 2012) package for the R programming language. Results from different PCA implementations should not differ; however, given the complexity and size of genetic data, specialized bioinformatic software such as PLINK is usually preferable to more generic statistical software.

## 4 Quality Control

In addition to estimating structure within the samples in our study, we also need to identify the individuals and genomic markers that are appropriate for our study. The first set of criteria that we can use to select our data corresponds to the task of spotting genotyping errors. These criteria are discussed in more depth in several reviews, as well as in original published research, and include missingness (applied to SNPs and samples), case-control differential missingness and tests for heterozygosity and Hardy-Weinberg equilibrium, and strand alignment checks when multiple datasets are involved (Turner et al., 2011; Medina-Gomez et al., 2015). Quality control is of particular importance when combining data from several sources in order to avoid confounding batch effects. However, there are some caveats that need to be considered when applying these criteria, because even though they are standard in homogeneous randomly mating populations they may not be appropriate in structured populations.

• Missingness. This includes removing SNPs that may give misleading results due to genotyping errors across many samples, or samples that have an excess of errors in the genotyping process and too few high-quality SNP.
• Strand alignment. Since DNA is double stranded, it is important to report (and compare) equivalent strands in the data; this can be a problem when merging data from different sources since there can be discrepancies in the reported strand. For example, the Illumina platform differs in definition on the concept of strand from the standard human genome reference (Zhao et al., 2018). It is important then to align the samples to the same strand. This becomes specially difficult in circumstances such as when the strands have complementary alleles (AT/CG). If these kind of uncertain SNPs are not too frequent in the data, it is probably better to remove them, since they can bias the results.
• Heterozygosity. In a homogeneous randomly mating population, very high or low levels of heterozygosity can indicate poor quality genotyping. However, this test is not appropriate in a non-randomly mating population, because population structure can lead to extremes of heterozygosity (Boca et al., 2020).
• Deviation from Hardy-Weinberg (HW) equilibrium. This test, standard in population studies, evaluates whether the expected relationship between allele frequency and genotype frequency exists. However, HW equilibrium assumes that there is random mating in the population under study; so if there are clear subpopulations (different ancestries) the conditions are not met and the test is not valid as a criteria for assessing quality. Therefore, this test is not generally recommended to use directly when studying structured populations. If the populations are labeled (e.g. we have data from different, clear sources) then it is better to apply HW tests separately.

The second set of criteria we can evaluate with genotype data can elucidate the ancestry of the individuals in the study. For this set we can use the methods we described above: admixture analysis and principal component analysis. There are two ways in which these are used as part of quality control:

• Firstly, individuals whose ancestry is not well represented in either cases or controls should be removed. In the case of a continuous trait this is equivalent to removing outliers. This avoids ancestry-specific biases in the association test, but is not expected to affect the variability of the data ancestry-wise.
• Secondly, if distinct populations (e.g. African, European, Asian) are represented across the phenotype, the study can be partitioned over these distinct populations. This would allow us to obtain multiple association tests, the results of which can subsequently be combined (see the post-GWAS Interrogation section). This method reduces ancestry-related variability and bias of each of the studies but decreases the amount of data in each of them, diminishing the statistical power of each test.

In case-control studies in particular, the selection of controls is a crucial step. If there is a factor that can influence the outcome (in our case the phenotype) in some way other than the variable that we are measuring (the genotypes), then it must be accounted for in the experimental design. As an illustrative example, in a trial for testing a new drug, there may be covariates (such as sex or age) that should be controlled for in order to ensure that the effect of the drug versus a placebo is measurable; e.g. age and sex may influence the outcome variable due to, for example, metabolism changes and hormone differences. One option to control for these covariates is randomizing which patients will receive the drug. What this procedure does is ensure that the distribution of age and sex between cases and controls is effectively the same, so the influence of these variables does not influence our observation of the drug effects. In GWAS studies, the distribution we want to keep consistent between cases and controls (or across the continuous trait) is the ancestry. In the following example we will use the first two principal components to illustrate this.

### 4.1 A Motivating Example

**FIGURE 4:**
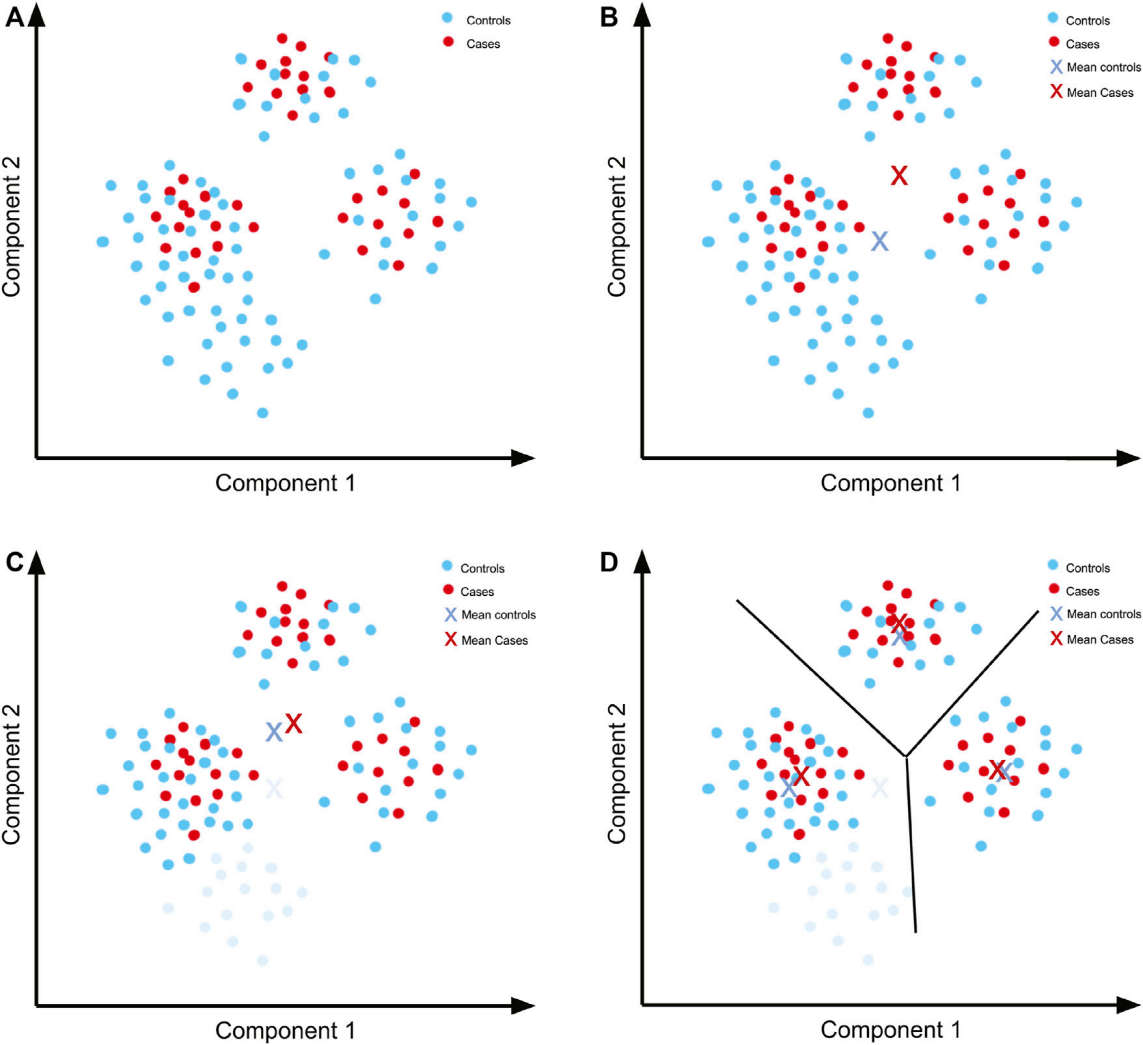
Quality control previous to performing a GWAS. **(A)** Hypothetical PCA plot for a simple dataset. There are three separate populations including both cases and controls. **(B)** The means of the components for cases and controls are not close because there is a concentration of controls in an area with no representation from cases. **(C)** By removing the controls that are not well represented in the cases (light blue), we can get a mean that is closer to the mean of the cases. **(D)** The means are closer when separating by the observed populations, so these can then be analysed separately and the results subsequently combined.

In order to develop a feeling for what quality control means in a GWAS, imagine a simple dataset (Figure 4A) to which PCA was applied and for which only the first two components are relevant to account for population structure.

Since the principal components represent a factor that we want to control (ancestry/ethnicity), we need a similar distribution of the components in both the cases and controls. We can further simplify the example by summarising the distribution using the mean (Figure 4B). Even by using only the mean of the data, it is evident that the distribution of controls does not follow the same distribution as the cases. A simple solution is to remove the controls with components that are unrepresented in cases (Figure 4C). The means of the cases and controls are now more similar, although not identical. We can further seek a better fit of the distributions by separating the populations according to the clusters that we can see in the plot.

Once this cluster separation has been done, there is a better fit in the distributions in each of the three sets of cases and controls (Figure 4D). Although for each of the association tests there will be less data to work with, and so less statistical power for each test, we can overcome this issue later via meta-analysis.

### 4.2 Comparability of Cases and Controls

**FIGURE 5:**
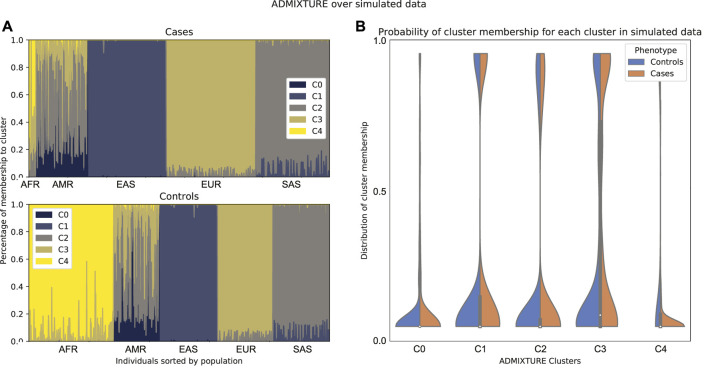
Comparison between admixture of cases and controls of simulated data according to a run of ADMIXTURE with K = 5. In these data, there is a super population that is underrepresented in the cases. **(A)** Admixture plot sorted by population label on 1,000 Genomes. **(B)** Density plot for cases and controls on the probability of membership to each of the clusters of the ADMIXTURE run. Note that here, cluster c4 corresponds to the African population.

In order to illustrate this approach, we up-sampled 2000 individuals from the 1000 Genomes Project dataset, removed a number of genotypically similar samples and assigned a fictitious case-control status to each of these in order to make the usefulness of the method more obvious. An ADMIXTURE run on the data shows a cluster that is underrepresented in the cases (Figure 5). This means that there is a super population from which almost no cases with the phenotype were sampled. In this case, it is a good idea to remove from the data the individuals from that population.

**FIGURE 6:**
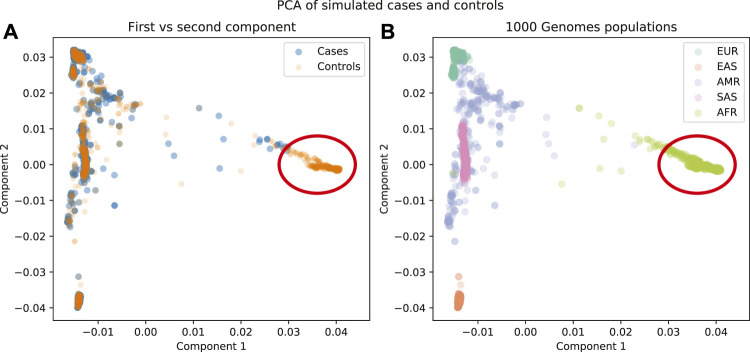
**(A)** First components plotted for cases and controls. **(B)** First components plotted for 1000 Genomes population labels. There is a region in these data that is not well represented in the cases as well as in the controls (red circle).

A PCA run on these data shows that there is a region of the plot where there are no cases, so the appropriate step would be to remove the individuals from that region (Figure 6). It is notable that if we just used the cluster results from the admixture analysis, cluster c4 would be a candidate for removal, but with PCA we find more nuanced criteria for the decision.

### 4.3 Separating the Data for Multiple Association Studies

If the clustering (in admixture analysis) or the separation (observed in PCA) is clear, such as in our sample data, it is preferable to analyse the populations in separate datasets as they may have different patterns of linkage disequilibrium. This can be useful to make statements about SNPs associated to the phenotype that are specific to subpopulations. However, if there are some population-specific signals for the tested trait, they may be lost in the subsequent meta analysis. If there are no distinct clusters, it is considered better to analyse the combined data in the association test (Begum et al., 2012).

**FIGURE 7:**
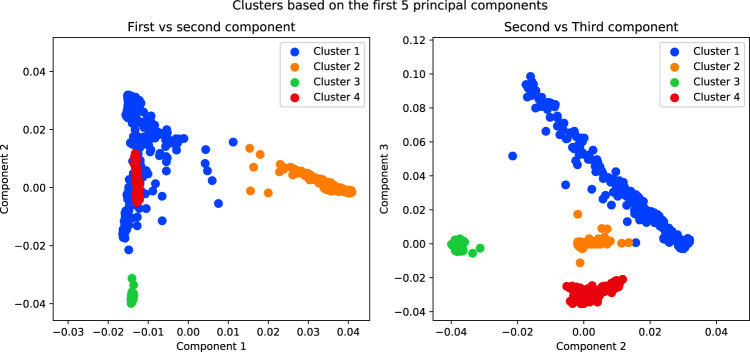
Results of running a K-Means algorithm on the first 5 components of the simulated data with K = 4. The purpose of this analysis is to separate the data into subpopulations in order to conduct association tests within each.

In order to separate the data, in admixture analysis we can choose for each individual the cluster for which the probability of membership is maximised as its cluster. For PCA, we can use a clustering method on the first *n* components (Figure 7).

## 5 Association Test

Once we have performed quality control of the samples and SNPs, and have chosen those to include in the analysis, as well as the number of separate population clusters we will be analysing, then we are ready to proceed to the identification of SNPs that are associated with the phenotype being tested. There are several ways to find candidate causal SNPs from genotype data, such as hypothesis testing and linear model-based approaches. In order to account for population structure, linear models are most widely used.

### 5.1 Methods to Perform Association Testing

#### 5.1.1 Logistic Regression

In the case of case-control studies, phenotypes are binary, and so we can use logistic regression. This model consists on assuming a linear relationship between independent variables and the log-odds, which represents the logarithm of the ratio of the probability of being a case over the probability of being a control conditioned on the covariates. That is, for two independent variables *x*_1_ and *x*_2_,

$$\log\frac{p}{1-p}=\beta_{0}+\beta_{1}x_{1}$$ (1)

**FIGURE 8:**
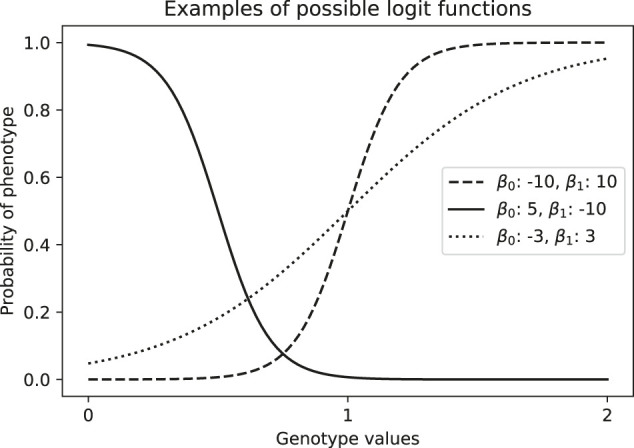
Examples of estimated probability of phenotype given a genotype value, which is coded numerically as the number of *alternative* alleles (aa = 0, Aa = 1, AA = 2).

Where *p* is the probability of being a case, *β*_0_ is the intercept and *β*_1_ is the effect size for the covariable *x*_1_. If we have more than one covariable, we can add more terms *β*_2_ *x*_2_, *β*_3_ *x*_3_, … . The model in Eq. 1 can yield results such as we see in Figure 8.

In any case, the logistic regression is performed on a locus-by-locus basis. This yields parameters with its respective *p*-value for each SNP. We will now discuss two methods to control for ancestry in the association test: via PCA and via admixture mapping. The difference between these methods lies in what independent variables are used in the logistic regression.

#### 5.1.2 Mixed Models

Mixed models are an extension of linear models that allow us to include effects that account for dependency between data points. For example, in the case of genetic studies, the data points are the individuals, and the dependency can be thought of as being the ancestry.

The model for a mixed effects regression for association testing can be written as follows

f(x)=Gβ+ν+Xγ (2)

Where the first term on the right side of the equation is the same as any linear model: the independent variables and the parameters; these are called the fixed effects and in the context of genetic association it is the genotype as described in the logistic regression section. The last term is the covariates (e.g., the first principal components, sex, etc). The second term represents the random effects, which model the error just like any other regression model, but in this case, the error is not equally distributed for every observation. Usually, we would say that the error follows a Normal distribution centered on zero with a fixed variance *N*(0, *σ* ^2^); but in mixed models we say that *ν* ∼ *N*(0, *τZ*), where *τ* is a parameter for *Z*, the matrix of random effects. *Z* is usually the genetic relationship matrix, which estimates the degree of sharing of identity by descent (IBD) between all pairs of individuals in the dataset, but it can also be a matrix of categories where each row (sample) is a vector of zeros everywhere except in the columns that represent the subpopulation to which it belongs (e.g. from Admixture analysis).

This is a general definition of mixed models, but there are several particular implementations based on variations of Eq. 2 and in particular of matrix *Z* such as EMMA (Kang et al., 2008), FaST-LMM (Lippert et al., 2011), GCTA-LOCO (Yang et al., 2014) and some Bayesian modelling versions like BOLT-LMM (Loh et al., 2015).

### 5.2 Controlling the Association Model for Ancestry

In the association test, we can model each locus as an independent variable with values 0, 1 or 2 depending on the dosage of the alternative allele (aa, Aa, AA respectively) with the trait being measured as a dependent variable. This model allows us to add other covariables; in particular, we can use the first principal components from the genotype PCA. Since the components absorb information about the ancestry, the model will only give significance to the SNPs that are related to the trait without the confounding of the population structure captured by the PCs included in the model.

**FIGURE 9:**
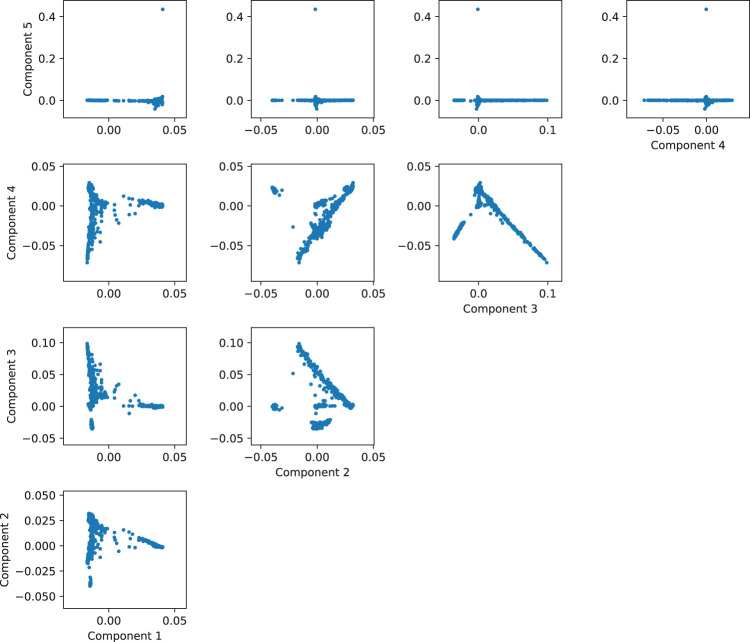
Matrix of scatterplots between the first five principal components of simulated data based on 1,000 Genomes.

One way of determining how many components to use consists in plotting the components until no separation is found in the data. In our 1000 Genomes example, there is clear separation of the individuals in the scatterplot between components one through four, but the direction of the fifth component is reaching for a subset of less than 1% of the data (the few points with the component 5 greater than 0.4), so it is not accounting for a significant amount of ancestry-related variability (Figure 9). So in this case, we would probably be safe in controlling by using only the first 4 components. This is a simple example, but it is useful in practice to visually review the interaction between the components to get a grasp of the structure of the data. For a more automated and statistically sound procedure, the software EIGENSOFT provides methods to infer the statistically significant number of components for population structure by evaluating the significance of each component iteratively according to the variance explained by each (Patterson et al., 2006).

An example of this usage can be found in Nannini et al. (2017). The authors use PCA to compare their Latino population with a reference panel of Europeans and Africans. They also determine that using four principal components in their regression is enough to control for population stratification. Another interesting example can be found in Costa-Urrutia et al. (2019), where authors control not by the principal components, but for the proportion of Amerindian ancestry estimated via ADMIXTURE.

As for local ancestry, in Wang et al. (2011) the authors propose controlling each of the tests by their respective estimated local ancestry. However, this method is not widely used, as it has been argued that the bias introduced by using only global ancestry is small (Martin et al., 2018). The methods that we discuss below exploit the advantages of local ancestry more directly.

### 5.3 Admixture Mapping

Admixture mapping is motivated by the scenario of recent mixing of populations which occurs alongside discrepant incidence of the trait between two populations (i.e. a difference in the proportion of affected people between the ancient populations). Affected persons in the admixed population should therefore be expected to have preferentially inherited the risk locus from the higher incidence population (Patterson et al., 2004). The genome-wide approach is to examine each region of the genome systematically to identify regions where affected persons inherit a statistically higher proportion of their alleles from the high risk population than the overall pattern of inheritance for that person.

This method relies on the assumption that the phenotype-associated alleles have different frequencies across ancestral populations. This extra requirement helps specify a model with more statistical power to find these specific loci, so in this way fewer SNPs (and since this implies lower burden of tests, also fewer samples) are needed to find associations. However, this means that it will fail to identify all risk loci; since not all causal SNPs follow this pattern. Also, fewer loci means longer LD tracts and so a higher difficulty in identifying causal markers via fine mapping (Seldin, 2007).

Admixture mapping has been successfully used to identify risk loci associated with specific ancestries across different traits; the tools and panels necessary for performing these kinds of analyses were developed in early 2000s. In 2005, the first applications of this seminal method were published, focusing on the study of African American individuals and finding a number of ancestry-specific associated loci (i.e., either European or African) to the traits: Zhu et al. (2005) found that excess African ancestry at 6q24 and 21q21 was associated with hypertension, and Reich et al. (2005) identified a European-derived locus in chromosome 1 associated to multiple sclerosis. Later, Freedman et al. (2006) identified that excess African ancestry at the 8q24 locus is associated to increased risk of prostate cancer.

More recently, Wang et al. (2019), used admixture mapping to find loci related to several traits used to measure sleep apnea; this study was performed on Latinos and found three novel regions associated with this condition. In another study in the Latino population, Burkart et al. (2018), identified genomic regions associated with lung function and chronic obstructive pulmonary disease, some of them previously undiscovered. In both of these studies, some of the risk loci found were replicated in Europeans, which illustrates the advantage of using samples from admixed populations.

As mentioned above, ADMIXTURE and STRUCTURE take different approaches to estimate a person’s proportion of genome inherited from an ancestral population (global ancestry). If, as computed using either of these approaches, the average proportion of genome from the higher risk population is estimated as *θ* for a study participant, then the genome-wide analysis is conducted for each participant by examining their actual inheritance at each SNP from this average across the genome. The calculation of the actual number of alleles at this SNP that have ancestry from the high risk subpopulation requires some discussion (local ancestry). Analysis of a single SNP will often be uninformative in terms of identifying the ancestral origin of each allele so instead the approach required is to use SNPs in proximity to the SNP under consideration to estimate the actual number of alleles from the high risk subpopulation (McKeigue, 1998).

If *x* is the estimated number of alleles at an SNP that have ancestry from the high-risk subpopulation (0, 1, 2) for a person, then given *θ* and *p*, the prevalence of the disease (0.5 with equal number of cases and controls), we can fit the logistic regression model from Eq. 3 (Hoggart et al., 2004):

$$\log\frac{p}{1-p}=log\frac{\pi }{1-\pi }+\left(\frac{x}{2}-\theta \right)\beta$$ (3)

Where *β* is the odds ratio for having 2 copies of the risk allele versus 0 in the high risk population. In the formula, the left hand term is the log odds of the trait. The right hand term of the equation has two components: the first one reflects the prevalence of the disease in log odd terms, and the second models genetic risk considers deviation from the average genotypic contribution from the high risk population for that person.

One extra advantage of admixture mapping is that, since this model examines ancestry at each SNP with the average across the genome for that person, there is an alternative test that can be done without controls (the so called “case only study”). It involves testing whether there is an increased risk according to the local ancestry in a given SNP. However, in practice, power is usually greater for the case-control comparison.

One widely used software to run admixture mapping can be found in the GENESIS package for the R programming language via the admixMap function.

### 5.4 Local Ancestry Regression

A novel approach is using the inference of local ancestry directly in the association testing. The software Tractor (Atkinson et al., 2021) implements the following regression model for each locus:

$$\log\frac{p}{1-p}=\beta_{0}+\beta_{1}X_{1}+\beta_{2}X_{2}+\beta_{3}X_{3}+\cdots +\beta_{k}X_{k}$$ (4)

Where every *β*_*i*_ are the effect estimates, *X*_1_ is the admixture proportion from the first ancestry, *X*_2_, *X*_3_ are the number of copies of the *alternative* allele coming from the first and second populations respectively (aa = 0, Aa = 1, AA = 2), and after that we can add any number of covariates such as age or some PCA components. This model allows for the inclusion of ancestry specific information, and in that way it results in relevant summary statistics related directly to each of the populations of the admixture. This model accounts only for two ancestries, however the model can be expanded to several ancestries.

Having a parameter associated to the ancestry in a given locus prevents the association model from attributing an effect to the allele count that is better explained by the ancient population from which the haplotype is descended. This avoids bias caused by local ancestry differences between populations that are not attributable to the trait (Atkinson et al., 2021).

In addition, although this method is analogous to controlling via PCA in the sense that we are controlling for ancestry, this type of regression achieves this by analyzing the ancestry of each specific locus at a time. This allows us to add samples without worrying about introducing population structure, which then translates into more statistical power.

These ancestry specific parameters provide information on ancestry predisposition to the trait. In contrast to admixture mapping, this method does not assume that the phenotype incidence differs across the ancestral populations. In this model, however, it is necessary to have data on both cases and controls.

## 6 Post-Genome-Wide Association Studies Interrogation

Association tests are performed on a SNP by SNP basis, so after the candidate SNPs have been identified, it is important to use techniques that help us validate the adequacy of our population adjustments in the previous steps. The technique of genomic control will allow us to evaluate whether the association test has a bias based on population structure. Performing a meta-analysis will allow us to combine the results of the different populations if we previously decided to separate by subpopulations in the quality control step.

### 6.1 Genomic Control

This method corrects the test statistics (*p*-values) obtained from the association analysis based on a single number, usually called the genomic inflation factor (Pritchard and Rosenberg, 1999) and denoted as *λ*. The inflation factor is calculated using the genetic markers that are not related to the disease, and it consists in testing whether there is a consistent difference between the allele frequencies in cases and controls across the genome.

This factor can be interpreted as follows: If *λ* = 1, there is no population stratification, and values greater than 1 indicate that there is structure unaccounted for in the study. However, in large well-powered studies, the inflation that this factor measures could be coming from a different source, such as polygenicity. For a more nuanced approach we can use LD score regression (Bulik-Sullivan et al., 2015), which leverages the relashionship between the SNP in question and those around it to discriminate the source of the inflation.

Even though the inflation factor can be used to correct for population stratification, it is not generally recommended to do so (Shmulewitz et al., 2004), as it is particularly ineffective in highly admixed data. It is however useful for identifying the presence of inflation in order to evaluate whether the methods in previous steps of the analysis were sufficient to account for population structure (Galanter et al., 2014; Conomos et al., 2016; Hodonsky et al., 2017; Jorgenson et al., 2017; Nannini et al., 2017).

### 6.2 Meta-Analysis

The meta-analysis is not in itself a method for correcting for population structure, but it is employed to analyse GWAS results from different populations. If we used the methods discussed in the Quality Control section to separate our individuals and performed one association test for each of those subpopulations, we can perform a meta-analysis to aggregate their results. This will help us regain statistical power lost by the reduced sample sizes of each study; the power is of course reduced if the effects are specific to some subpopulation, and this will be true no matter the analytical approach.

The results that we intend to aggregate from the studies are the effect sizes (*γ*) for the trait. However, since factors such as sample size can influence the existence of different levels of uncertainty on each study, we must have a measure available to assess uncertainty. For this purpose, having also the standard error will allow us to perform an inverse variance-weighted meta-analysis; which means that we are using the variance of the estimator to weigh in the uncertainty found in each of the studies before performing the meta-analysis.

The first model we can use is to use a fixed-effects-only model. This assumes that all of the effect sizes across all studies are the same, and the differences between them are the product of a normally distributed random error (*ϵ*).

γ=β+ϵ (5)

Another possible model would be to use a random-effects-only model. This is applied when we suspect that the underlying effect size varies between studies, for instance due to different patterns of linkage disequilibrium or gene-environment interactons.

$$\gamma =\theta +\mu_{i}+\epsilon$$ (6)

Where *θ* is the true effect size, and *μ* is the within study variance that will be estimated from the data (Kelley and Kelley, 2012).

The difference between the two models then, is that in the fixed effects model we are assuming that there is a single, true effect size across all the studies, and we are trying to find whether this true effect size is different from zero. In the random effects model we are assuming that there is a distribution of random effects, and we are trying to find whether the mean of the effect sizes is different from zero.

The fixed effects model assumes that there is no heterogeneity between the effects in the different studies being combined, this can be tested by referring to Higgins and Thompson (2002), where they propose a metric *I* ^2^ that measures the proportion of variation between studies that is due to heterogeneity. They propose as a rule of thumb that with an *I* ^2^ > 30% we should consider using random effects instead of fixed effects. The fixed effects model provides more power, but it is important to examine its appropriateness before enjoying its benefits.

Jorgenson et al. (2017) provide an example of a study with different ethnicities (Non-Hispanic Whites, Hispanic/Latinos, East Asians, and African Americans) where authors decided to separate the analysis into different studies and used meta-analysis to aggregate the results. They were successful in describing both genotype-phenotype associations that were unique to individual populations, and signals that reached significance when all populations were taken into account via a trans-ethnic meta-analysis.

If we have been careful in performing all steps above, including quality control, association testing and post-GWAS interrogation, we should have a list of SNPs that are enriched for real genotype-phenotype associations.

## 7 Discussion

In this review, we have attempted to give an overview of the methods used for performing GWAS on admixed populations. The main objective was to shed some light on the intuition behind using each of them.

1) Quality Control. The objective in this step is to remove low-quality SNPs and samples and to ensure a comparable population structure across the phenotype (e.g. same distribution among cases and controls).
• Comparability of cases and controls. Removing outliers from the data can be convenient to the analysis, but excluding whole subpopulations hurts the generalizability of the study. This strategy is used mostly when the control data has not been sampled according to the same protocol as the cases, like the case of using a generic database such as a biobank.
• Separating the data for multiple association studies. If there is an overrepresentation of a subpopulation or if there is a need to report population specific related SNPs, it could be convenient to analyse the data separately. The main caveat of doing this is the possibility of having to perform an association test with few data.
2) Controlling for ancestry at the association test step. Here, we account for population structure in the actual modeling of the genotype-genotype relationship. This helps avoid spurious correlations. Methods that we can use for this purpose are:
• PCA. There is no reason not to control for ancestry using PCA, but it is important to add the correct number of components to the model (Tian et al., 2008). The recommendation is to review the distribution of the data in several component plots and to examine the results of inflation by using the genomic factor, or use specialised software such as EIGENSTRAT.
• Admixture mapping. If there are no clearly distinct subpopulations found in the sample, admixture mapping is an appropriate way to find regions where the admixture is related to the phenotype. Some methods such as Tractor can also find the specific effect sizes on each of the subpopulations.
3) Post-GWAS interrogation. As in many other cases of experimental studies, the results of a statistical procedure should be analysed and should be open to correction according to the data and data cleaning that has been used.
• Genomic control. This tool is useful as a measure of the population structure that has been introduced to the study, and to suggest whether or not it is necessary to go back to previous steps in order to further account for the structure of the data. Although it is possible to use it to control for overall population structure by scaling the p-values of the association test, it is not recommended and should be used only as a sanity check.
• Meta-analysis. This is necessary in order to aggregate the results in the case that we have separated the data into its subpopulations. It is possible to achieve the same power as a whole-data association test given some properties, but any population specific signal that may have appeared in the individual studies might be lost in the meta-analysis.
