# Supplementary figures and images for: An Overview of Strategies for Detecting Genotype-Phenotype Associations Across Ancestrally Diverse Populations
Source: Front Genet. 2021 Nov 5;12:703901. doi: 10.3389/fgene.2021.703901 (PMC8602802; doi:10.3389/fgene.2021.703901)

# PCA of simulated cases and controls

## First vs second component

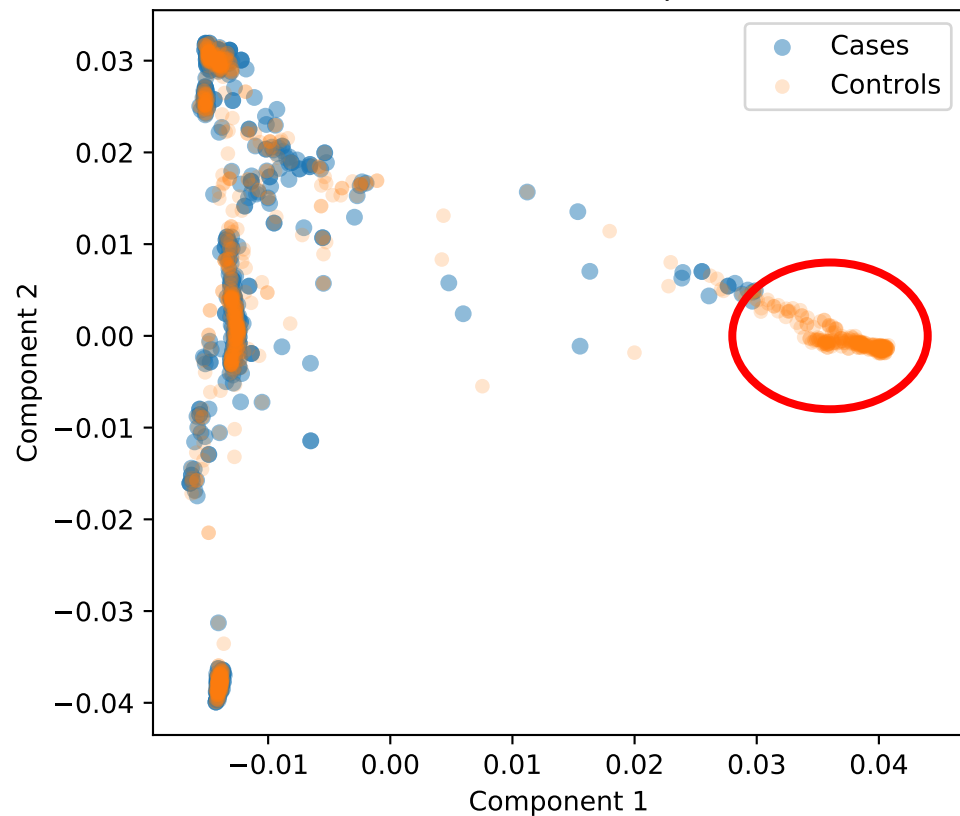

## 1000 Genomes populations

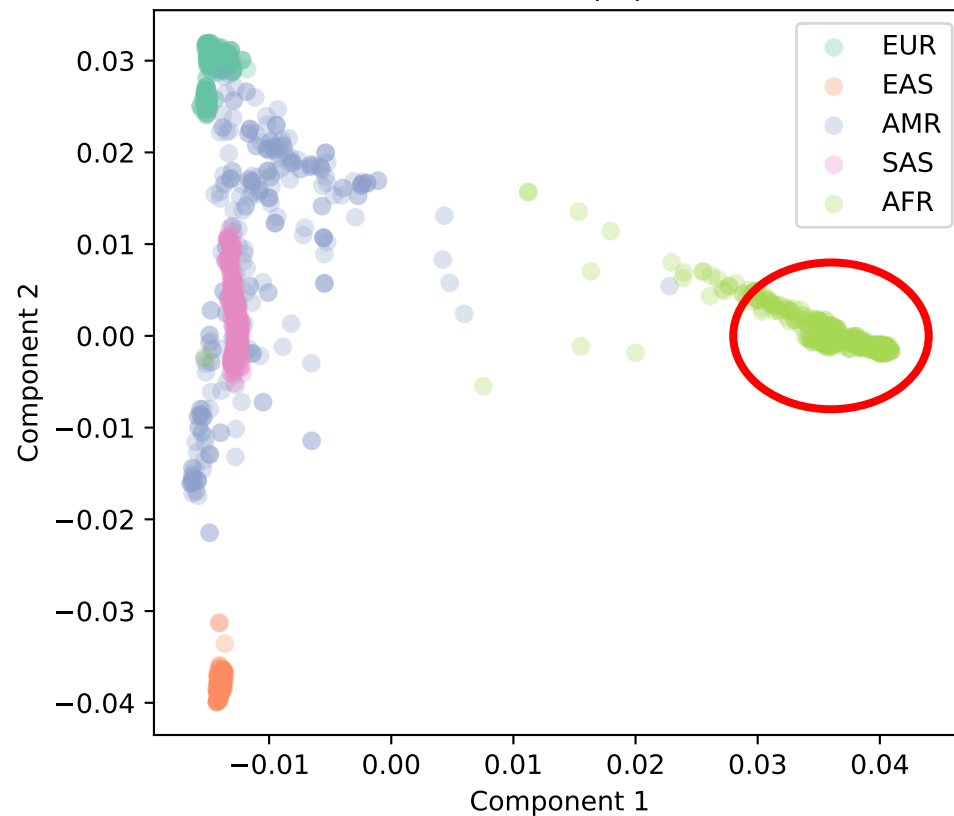

Supplement: Supplementary file 1 [file DataSheet2.PDF]
