# Supplementary material for: An Overview of Strategies for Detecting Genotype-Phenotype Associations Across Ancestrally Diverse Populations
Source: Front Genet. 2021 Nov 5;12:703901. doi: 10.3389/fgene.2021.703901 (PMC8602802; doi:10.3389/fgene.2021.703901)
Supplement: Supplementary file 5 [file DataSheet1.PDF]

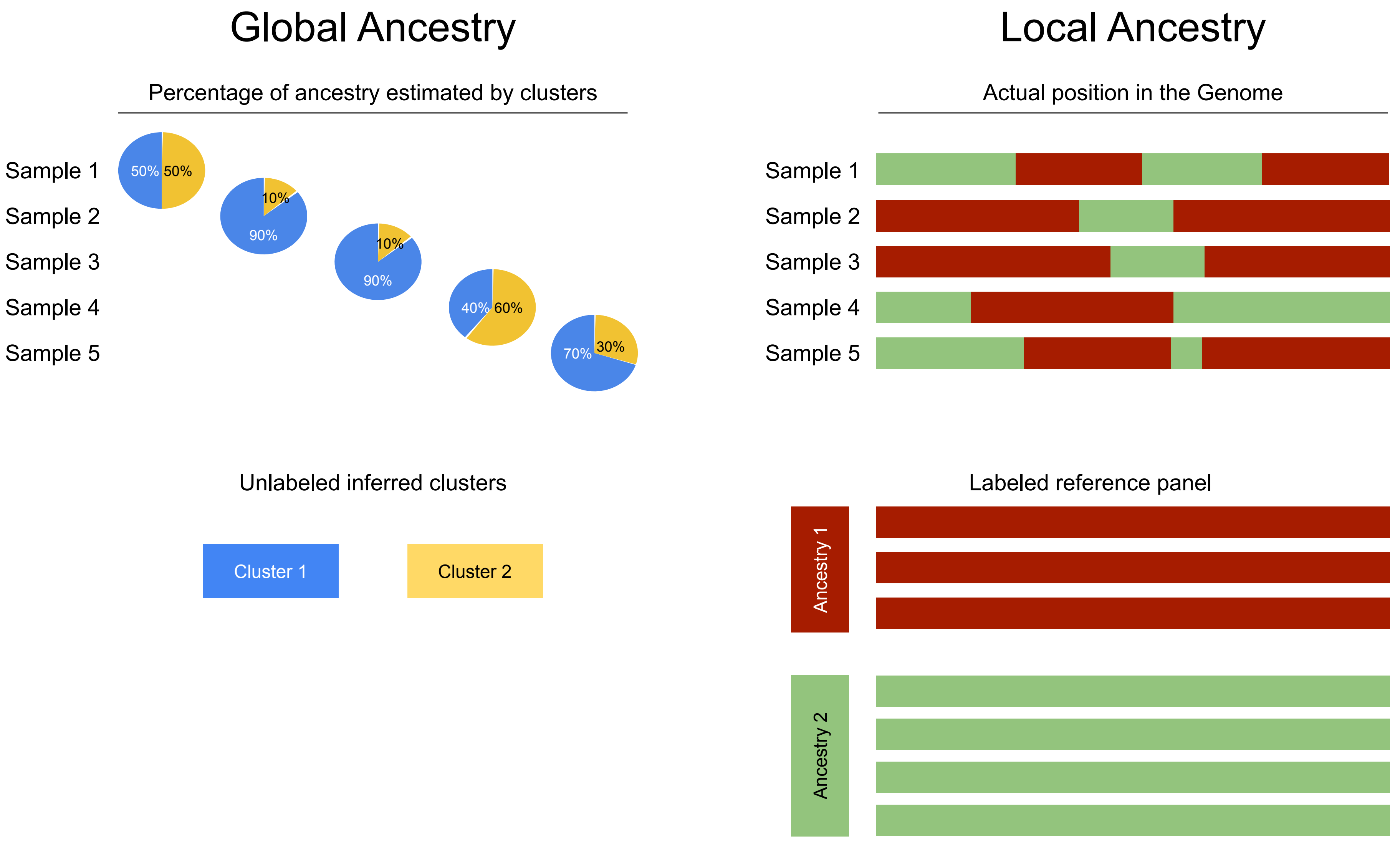
Global Ancestry
Percentage of ancestry estimated by clusters
Sample 1
Sample 2
Sample 3
Sample 4
Sample 5
50%
50%
10%
90%
10%
90%
40%
60%
70%
30%
Unlabeled inferred clusters
Cluster 1
Cluster 2
Local Ancestry
Actual position in the Genome
Sample 1
Sample 2
Sample 3
Sample 4
Sample 5
Labeled reference panel
Ancestry 1
Ancestry 2
